# Lyse-Reseal Erythrocytes for Transfection of *Plasmodium falciparum*

**DOI:** 10.1038/s41598-019-56513-9

**Published:** 2019-12-27

**Authors:** Gokulapriya Govindarajalu, Zeba Rizvi, Deepak Kumar, Puran Singh Sijwali

**Affiliations:** CSIR-Centre for Cellular and Molecular Biology, Hyderabad, 500007 TS India

**Keywords:** Transfection, Parasitic infection

## Abstract

Simple and efficient transfection methods for genetic manipulation of *Plasmodium falciparum* are desirable to identify, characterize and validate the genes with therapeutic potential and better understand parasite biology. Among the available transfection techniques for *P. falciparum*, electroporation-based methods, particularly electroporation of ring-infected RBCs is routinely used. Nonetheless, transfection of *P. falciparum* remains a resource-intensive procedure. Here, we report a simple and economic transfection method for *P. falciparum*, which is termed as the lyse-reseal erythrocytes for transfection (LyRET). It involved lysis of erythrocytes with a hypotonic RBC lysis buffer containing the desired plasmid DNA, followed by resealing by adding a high salt buffer. These DNA-encapsulated lyse-reseal erythrocytes were mixed with *P. falciparum* trophozoite/schizont stages and subjected to selection for the plasmid-encoded drug resistance. In parallel, transfections were also done by the methods utilizing electroporation of DNA into uninfected RBCs and parasite-infected RBCs. The LyRET method successfully transfected 3D7 and D10 strains with different plasmids in 63 of the 65 attempts, with success rate similar to transfection by electroporation of DNA into infected RBCs. The cost effectiveness and comparable efficiency of LyRET method makes it an alternative to the existing transfection methods for *P. falciparum*, particularly in resource-limited settings.

## Introduction

*P. falciparum* causes the most virulent form of malaria and is responsible for the majority of malaria-associated mortality. Lack of a vaccine and continued emergence of drug resistant strains are challenges to malaria control and elimination efforts. Limited understanding of the biology of *P. falciparum* is also a barrier to the identification of targets for development of new chemotherapeutics and diagnostics. The availability of genome sequences of *P. falciparum* and other *Plasmodium* species serves as a treasure to be unlocked for understanding the complex, yet interesting, biology of *Plasmodium*. Manipulation of desired genes or gene products using reverse genetics techniques has been immensely useful in studying model organisms. Early transfection methods for *Plasmodium* utilized electroporation of plasmid DNA into asexual blood stage-infected erythrocytes^1–5^. Attempts have been made to improve transfection efficiency by electroporation of DNA into uninfected erythrocytes followed by infection of these DNA-preloaded erythrocytes by schizont stage parasites (Epreloading method)^6^. Electroporation-independent procedures have also been used with varying success^7–9^. Nucleofection of the schizont stage of *P. berghei*, a rodent malaria parasite, greatly enhanced transfection efficiency and decreased selection period^10,11^. Nucleofection has also been adapted for high-throughput transfection of *P. falciparum*^12^. However, nucleofection requires expensive proprietary reagents and an electroporation device. Development of transfection procedures for genetic manipulations of *Plasmodium* species and the availability of *Plasmodium* genome sequences have led to several exciting findings on the parasite biology, including the recent reports of genome-wide gene knock outs in *P. falciparum* and *P. berghei*^13,14^. Electroporation of DNA into ring stage-infected red blood cells (EiRBC method) remains the most used method in the field at present^5^.

Transfection of *P. falciparum* is still considered a resource-intensive procedure. Since *P. falciparum* can take up plasmid DNA present in the erythrocyte cytosol and resealed erythrocytes remain infective to *P. falciparum*^6,15–18^, we combined these two properties to develop a transfection procedure. Erythrocytes were lysed with a hypotonic RBC lysis buffer containing the desired plasmid DNA, resealed by adding a high salt buffer, mixed with purified trophozoite/schizont stage parasites, and subjected to drug selection. We have named this method as the lyse-reseal erythrocytes for transfection (LyRET). The success rate of transfection of *P. falciparum* by LyRET was comparable with EiRBCs method and was higher than that of Epreloading method.

## Materials and Methods

*P*. *falciparum* 3D7 and D10 strains were obtained from the Malaria Research and Reference Reagent Resource Centre (MR4). RPMI 1640 (cat No. 12-115Q) was procured from Lonza. Hypoxanthine (cat No. 11067-030), albumax II (cat No. 11021037), gentamicin (cat No. 15750-060) and blasticidin S HCl (cat no. R210-01) were from Invitrogen. Restriction enzymes and DNA modifying enzymes were from New England Biolabs Inc. and Thermo Fisher Scientific. DNA isolation kits were from Qiagen and MACHEREY-NAGEL. All biochemical were from standard suppliers like Sigma and Serva. All the tissue culture plastic ware was from standard manufacturers such as Corning Inc, Nalgene and Tarsons. All experiments were performed in accordance with relevant guidelines and regulations.

### Parasite culture

Whole blood was collected from human volunteers after obtaining informed consent by venipuncture according to the approved protocols of Institutional Ethics Committee of Centre for Cellular and Molecular Biology (IEC-38/2015 and IEC-38-R3/2019). The blood was centrifuged at 662xg for 5 min at room temperature using a swinging bucket rotor; plasma and buffy coat were carefully removed by aspiration. The RBC pellet was washed twice, each time with 2x packed cell volume (PCV) of the RBC storage medium (RPMI 1640 with 2 g/l glucose, 300 mg/l glutamine and 25 µg/ml gentamicin). The RBCs were stored as 50% hematocrit in RBC storage medium at 4 °C until used. RBCs from different donors irrespective of the blood group were used during routine parasite culture and for the majority of transfection experiments, except for transfection experiments with individual blood group RBCs.

The *P. falciparum* strains were grown at 37 °C under a mixed gas environment (5% CO_2_, 5% O_2_ and 90% N_2_) in the parasite culture medium (RPMI 1640 with 2 g/l sodium bicarbonate, 2 g/l glucose, 25 µg/ml gentamicin, 300 mg/l glutamine, 0.5% albumax II) containing human erythrocytes at 2% hematocrit^19^. Parasites were synchronized by treatment with 5% D-sorbitol when the majority of parasites were at ring stage^20^. For purification of late trophozoite/schizont stages, synchronized ring stage culture (10–15% parasitemia) was grown till majority of the parasites reached late trophozoite/schizont stage. 25–30 ml of the culture was transferred to a 50 ml conical centrifuge tube, 10 ml of 65% nycodenz density gradient solution was carefully layered underneath the culture. The sample was centrifuged at 360 × g for 15 min at 25 °C using a swinging-bucket rotor (with maximum acceleration and zero deceleration). The interphase containing iRBCs was carefully collected and transferred to a 15 ml conical centrifuge tube. The suspension was centrifuged at 662 × g for 5 min at room temperature using a swinging-bucket rotor, the supernatant was carefully aspirated, the pellet was resuspended in 10 ml parasite culture medium and centrifuged at 662 × g for 5 min at room temperature using a swinging-bucket rotor. The supernatant was discarded and the pellet was washed again with the parasite culture medium. The final pellet was suspended in 1 ml parasite culture medium. An aliquot of the purified sample was processed for Giemsa smear to assess purity of the sample.

### Transfection plasmids

Three plasmids were used for transfection: pFCEN1, pPfCENv3 and HFDDI. pFCEN1 was a kind gift from Dr. Shiroh Iwanga^21^. It contains human dihydrofolate reductase (hDHFR) cassette for selection of recombinant parasites with WR99210 and GFP cassette for evaluation of recombinant parasites for GFP expression using fluorescence microscopy or western blotting. pPfCENv3 was derived by replacing the GFP coding sequence at AvrII-AflII sites in pPFCENv2 with another GFP coding sequence, which was amplified from the pSTCII-GFP using primers GFPcen-F (ATTACCTAGGAGATCTCAAAATGGGTACC; contains AvrII/BglII sites) and GFPcen-R (ATTACTTAAGCTCGAGTTAGGATCCCTG, contains XhoI/AflII sites)^21,22^. pPfCENv3 contains blasticidin S deaminase (BSD) and GFP cassettes for selection of recombinant parasites with blasticidin and evaluation for GFP expression, respectively. HFDDI was derived from the DJ1KO plasmid and contains hDHFR cassette that allows selection of transfected parasites with WR99210^23^. All the three plasmids were prepared using Qiagen or MACHEREY-NAGEL maxi preparation kits according to the manufacturer’s instructions. Plasmid DNA pellets were resuspended in RBC lysis buffer (5 mM K_2_HPO_4_, 1 mM ATP, pH 7.4; for LyRET method) or nuclease free water (for electroporation methods).

### Preparation of lyse-reseal erythrocytes

The RBC suspension (~200 μl/experiment) was transferred into a 1.5 ml microcentrifuge tube (MCT) and centrifuged at 371 x g for 5 min at 4 °C using a fixed-angle rotor. The pellet (~100 μl PCV) was washed twice, each time with 1 ml ice cold PBS, and the supernatant was carefully removed. The pellet was resuspended in 100 μl of ice cold RBC lysis buffer (5 mM K_2_HPO_4_, 1 mM ATP, pH 7.4) and incubated at 4 °C for 1 hour with gentle rotation^15–18^. The lysed RBC ghost suspension was resealed as has been previously described with minor modifications^16^. The volume of RBC ghost suspension was estimated and appropriate volumes of solution stocks (5 M NaCl, 1 M MgCl_2_, 100 mM ATP, 100 mM GSH) were added to achieve the resealing buffer concentration (150 mM NaCl, 5 mM MgCl_2_, 1 mM ATP and 1 mM GSH). This suspension was incubated at 37 °C for 1 hour with shaking at 55 rpm to allow resealing of RBC ghost. The resealed RBC suspension was washed twice, each time with 10xPCV of RBC storage medium (prewarmed to 37 °C). The washed resealed RBC pellet was resuspended in equal volume of RBC storage medium for subsequent use or stored at 4 °C till used. The resealed RBCs were termed as the lyse-reseal erythrocytes (LREs).

For increased incorporation of the lysate protein content in resealed RBCs, the RBC ghost suspension was concentrated to 75% of its volume using a 3 kDa cut off centricon before resealing. Concentration was done at 4 °C and 2739 × g using a swinging-bucket rotor.

### Parasite growth in lyse-reseal erythrocytes

2.5 ml parasite culture medium containing normal erythrocytes or LREs prepared without or with the concentration step (all at 2% hematocrit) was inoculated with purified trophozoite/schizont stage parasites (1% final parasitemia). The cultures were grown for three consecutive cycles with the change of media and addition of fresh respective erythrocytes at the end of each cycle to maintain 2% hematocrit. Parasite stages and parasitemia were determined every 24 hours by making Giemsa smears and counting at least 1000 cells. This experiment was set up in triplicates and repeated upto three times.

### Transfection of *P. falciparum* using lyse-reseal erythrocytes

Detailed step-wise protocol is provided in the Supplementary Information. 100 μl packed cell volume of RBCs was washed twice with 1 ml ice cold PBS, lysed with ice cold RBC lysis buffer containing 100 μg plasmid DNA, and resealed as mentioned above in the “Preparation of lyse-reseal erythrocytes” section. LREs containing DNA were resuspended in 5 ml parasite culture medium (at 2% hematocrit), inoculated with purified trophozoite/schizont stages to achieve 2–3% parasitemia (day 0), and cultured under standard conditions. On the following day (day 1), the culture was fed with fresh parasite culture medium. On day 2, the culture was expanded to adjust parasitemia to 4–5%, and hematocrit was maintained at 2% by adding fresh normal RBCs. On day 3, selection for recombinant parasites was started by adding appropriate drugs in the parasite culture medium (blasticidin: 1 μg/ml for pPfCENv3; WR99210: 0.5–1.0 nM for HFDDI and pFCEN1). Cultures were grown under drug pressure for 5 consecutive cycles to eliminate non-transfected parasites, followed by in the absence of drug for 3 cycles to reduce stress to transfected parasites, and thereafter in the presence of drug. The culture medium was changed every day for the first week, followed by on alternate days, and 50 μl suspension of fresh normal RBCs was added to the culture once a week to replenish old and lysed RBCs. The cultures were routinely monitored for parasites by observing Giemsa smears. Upon emergence of recombinant parasites, the cultures were expanded and processed for downstream experiments like preparation of frozen stocks and evaluation of recombinant parasites.

pPfCENv3 was also transfected into RBCs of different blood groups (O+, A+, B+ and AB+) as has been described above. Briefly, 100 μg plasmid DNA was used to prepare LREs of each RBC group, trophozoite/schizont stage parasites were purified from a synchronized culture of *P. falciparum* 3D7 maintained in O blood group RBCs, and the RBCs of respective blood groups were used whenever fresh normal RBCs needed to be added to the culture.

### Transfection by electroporation of infected-RBCs

Transfection of ring stage-infected RBCs (EiRBC method) was performed as has been previously described with minor modifications^5,24^. Briefly, 5 ml culture of early ring stage-infected RBCs (5–10% parasitemia) was used for one transfection. The culture was centrifuged at 371 × g for 5 min at room temperature using a fixed-angle rotor, the pellet (~100 µl packed cell volume) was washed with cytomix (10 mM K_2_HPO_4_-KH_2_PO_4_, 120 mM KCl, 0.15 mM CaCl_2_.2H_2_O, 25 mM HEPES, 2 mM EGTA, 5 mM MgCl_2_.6H_2_O, pH 7.6) and then suspended in cytomix containing 50 µg plasmid DNA (final volume: 420 µl). The suspension was transferred to a chilled 0.2 cm cuvette and pulsed (at 0.31 kV, 960 µF and infinite Ω) using the Bio-Rad gene pulser Xcell™. The electroporated sample was immediately transferred to a flask with 5 ml parasite culture medium, and grown under standard conditions. 3–4 hours post-transfection, the culture medium was changed with fresh parasite culture medium (day 0). On day 1, the culture was expanded to 10 ml with fresh RBCs. Selection was started with the addition of appropriate drug to the culture on day 2. The parasite culture medium was changed every day for the first week, followed by on alternate days, and 50 μl fresh RBC suspension was added to the culture once a week to replenish old and lysed RBCs. The cultures were maintained thereafter as described above for the LyRET method.

pPfCENv3 was also transfected into RBCs of O blood group as has been described above. Briefly, early ring-stage infected RBCs of O blood group were electroporated with 50 μg plasmid DNA and the RBCs of same blood group were used whenever fresh normal RBCs needed to be added to the culture.

### Transfection by electroporation of DNA into uninfected RBCs

Electroporation of DNA into uninfected RBCs (Epreloading method) was performed as has been described earlier with minor modifications^6^. Briefly, uninfected RBCs (100 µl packed cell volume/transfection) were washed with cytomix, and then resuspended in cytomix containing 50 µg plasmid DNA (final volume: 420 µl). The suspension was transferred to a 0.2 cm cuvette and electroporated as described above for iRBCs method. The electroporated sample was transferred to a 15 ml conical centrifuge tube with 5 ml parasite culture medium, centrifuged at 662 × g for 5 min at room temperature using a swinging-bucket rotor. The pellet of DNA-preloaded RBCs was resuspended in 5 ml parasite culture medium, mixed with purified trophozoite/schizont stage parasites (2–3% final parasitemia), and grown under standard conditions (day 0). From day 1 onward, the culture was maintained as described for the LyRET method.

### Assessment of recombinant parasites

The pPfCENv3 and pFCEN1 transfected parasites were assessed for GFP expression using live cell fluorescence microscopy, and the HFDDI transfected parasites were assessed for the presence of plasmid DNA by PCR. For microscopy, a small aliquot of the culture was washed twice with PBS, stained with Hoechst (10 μg/ml), and immobilized on a poly L-Lysine coated slide for 20 minutes. Unbound cells were washed off with PBS and the slide was covered with a coverslip. The slide was observed under the 100x objective of ZEISS Axioimager microscope. Images were taken (Zeiss AxioCam HRm) and analysed with the Axiovision software. For PCR, the HFDDI transfected parasites were isolated from a 10 ml asynchronous culture (10–15% parasitemia) by saponin lysis. The parasite pellet was processed for genomic DNA isolation using the Puregene blood kit (Qiagen) as instructed by the manufacturer. Genomic DNA was used as a template for amplification of the *P. yoelii* α-tubulin region of HFDDI by PCR (primers: PyaTb5U-F: AGGGACCGGTGAAAAGCCCTAAATGC, HbTb-R: GACGATGCAGTTTAGCGAACCATGCATTTTTACTTGTATATTATAAAATAAACAATTG). Amplification products were resolved by agarose gel electrophoresis and stained with ethidium bromide.

Recombinant parasites obtained from the transfection of pPfCENv3 into different blood group RBCs were examined for GFP expression by live cell fluorescence microscopy and flow cytometry. The transfected cultures were maintained with the same blood group RBCs and grown for 5–6 cycles in the presence of blasticidin (1 μg/ml) after the cultures had reached to 1–2% parasitemia. 100 µl of each synchronized trophozoite stage culture was washed twice with PBS, the pellet was resuspended in 1 ml PBS containing Hoechst (10 µg/ml) and incubated at room temperature for 15 minutes. As a control, uninfected RBC sample (at 0.2% haematocrit) was also stained with Hoechst. The samples were examined using live cell fluorescence microscopy as described above. For flow cytometry, the samples were analysed on the Beckman Coulter Gallios A94303. Excitation wavelength was 405 nm for Hoechst and 488 nm for GFP. Emission was detected with FL9 channel (450/50) for Hoechst and FL1 channel (550 SP) for GFP. For each sample, a total of 1 × 10^6^ events were collected. The data was analysed using FlowJo and Kaluza softwares. The population of GFP positive cells was determined as percentage of the GFP-Hoechst positive cells in the total number of Hoechst positive cells.

## Results and Discussion

### Lyse-reseal erythrocytes support efficient parasite growth

**Figure 1:**
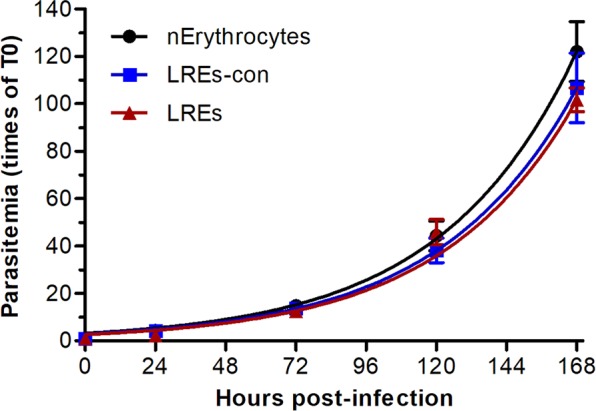
Growth profiles of *P*. *falciparum*. Purified trophozoite/schizont stage parasite were added to normal erythrocytes (nErythrocytes), LREs prepared without concentration (LREs) or after concentration (LREs-con) of the lysate. Parasite growth was monitored for 168 hours post-infection and parasitemia is shown as mean with SD from 3 experiments for nErythrocytes, 2 experiments for LREs-con and a single experiment for LREs.

This study was intended to develop an economical and simple method for transfection of *P. falciparum*. Although resealed erythrocytes have been shown to be infected by *P. falciparum*^15–18^, a quantitative comparison of parasite growth in resealed and normal erythrocytes is not available to our knowledge. Hence, we attempted optimization of LRE preparation to achieve maximum incorporation of the lysate protein content in resealed erythrocytes. The incorporation of protein content in resealed erythrocytes was ~53% of the total RBC lysate protein content. Concentration of RBC lysate to 75% of its initial volume increased incorporation to ~74% in resealed erythrocytes. The growth of *P. falciparum* in LREs prepared without or with the concentration step was compared with that in normal erythrocytes for 168 hours. The parasite growth was similar in both the LREs and normal erythrocytes for the first 120 hours, indicating that LREs supported efficient parasite invasion and development (Fig. 1). At the end of 168 hours of growth, parasitemia was marginally lower in LREs than that in normal erythrocytes, which could be due to lysis of LREs toward the end of assay. As the parasite growth in both types of LREs was similar, we went ahead with the LREs prepared without the concentration of RBC lysate, which eliminates the requirement of a concentration device and saves time.

### Successful transfection of *P. falciparum* by the LyRET method

**Figure 2:**
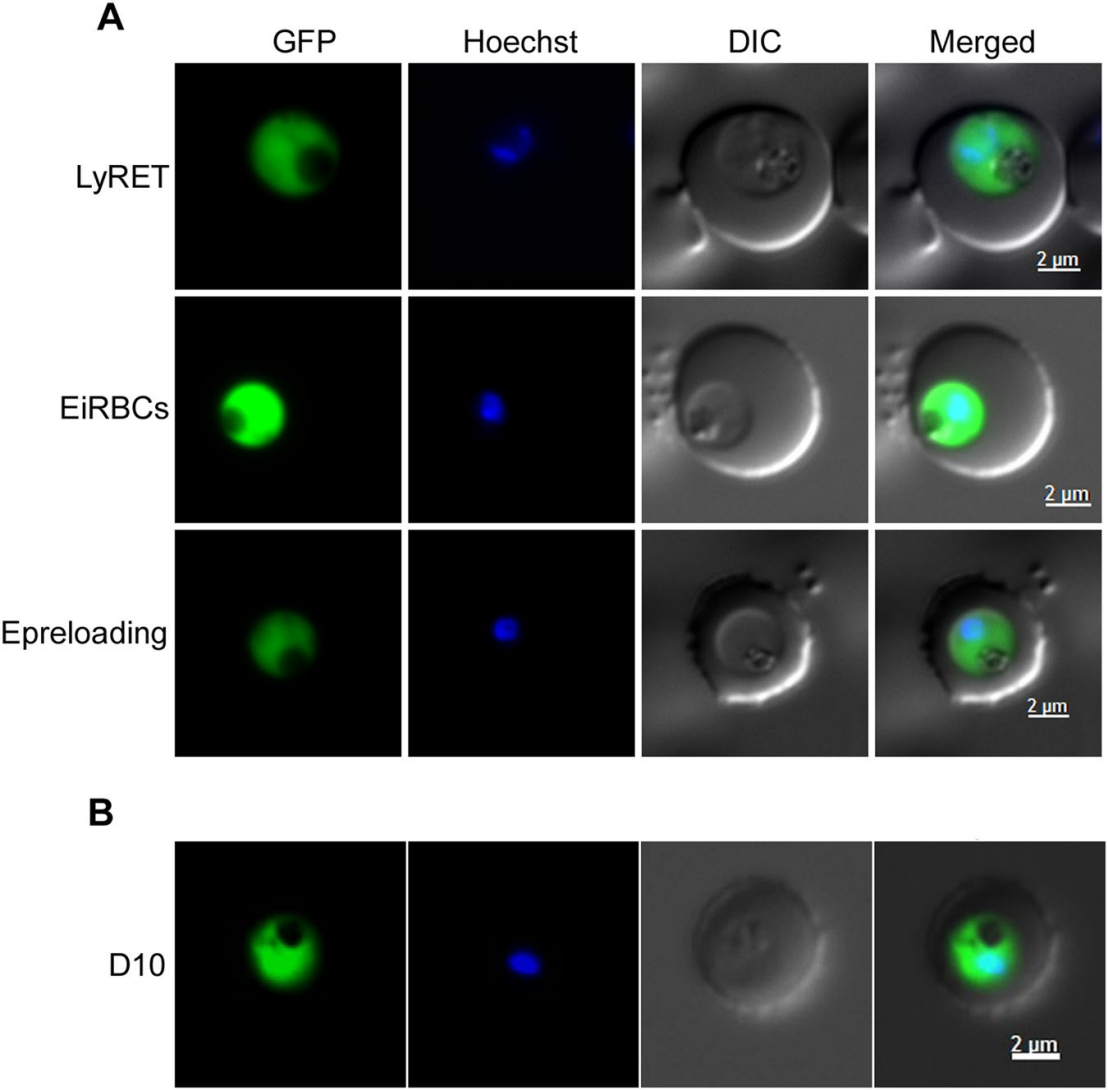
GFP expression in drug resistant parasites. (**A**) *P. falciparum* 3D7 was transfected with pPfCENv3 using LyRET, EiRBC and Epreloading methods. (**B**) *P. falciparum* D10 was transfected with pPfCENv3 using the LyRET method (D10). Drug resistant parasites were assessed for GFP fluorescence using live cell microscopy. Shown are the representative images for different methods. The panels in (A,B) are for GFP fluorescence in parasites (GFP), nuclear stain (Hoechst), bright field (DIC) and the overlap of all images (Merged).

**Figure 3:**
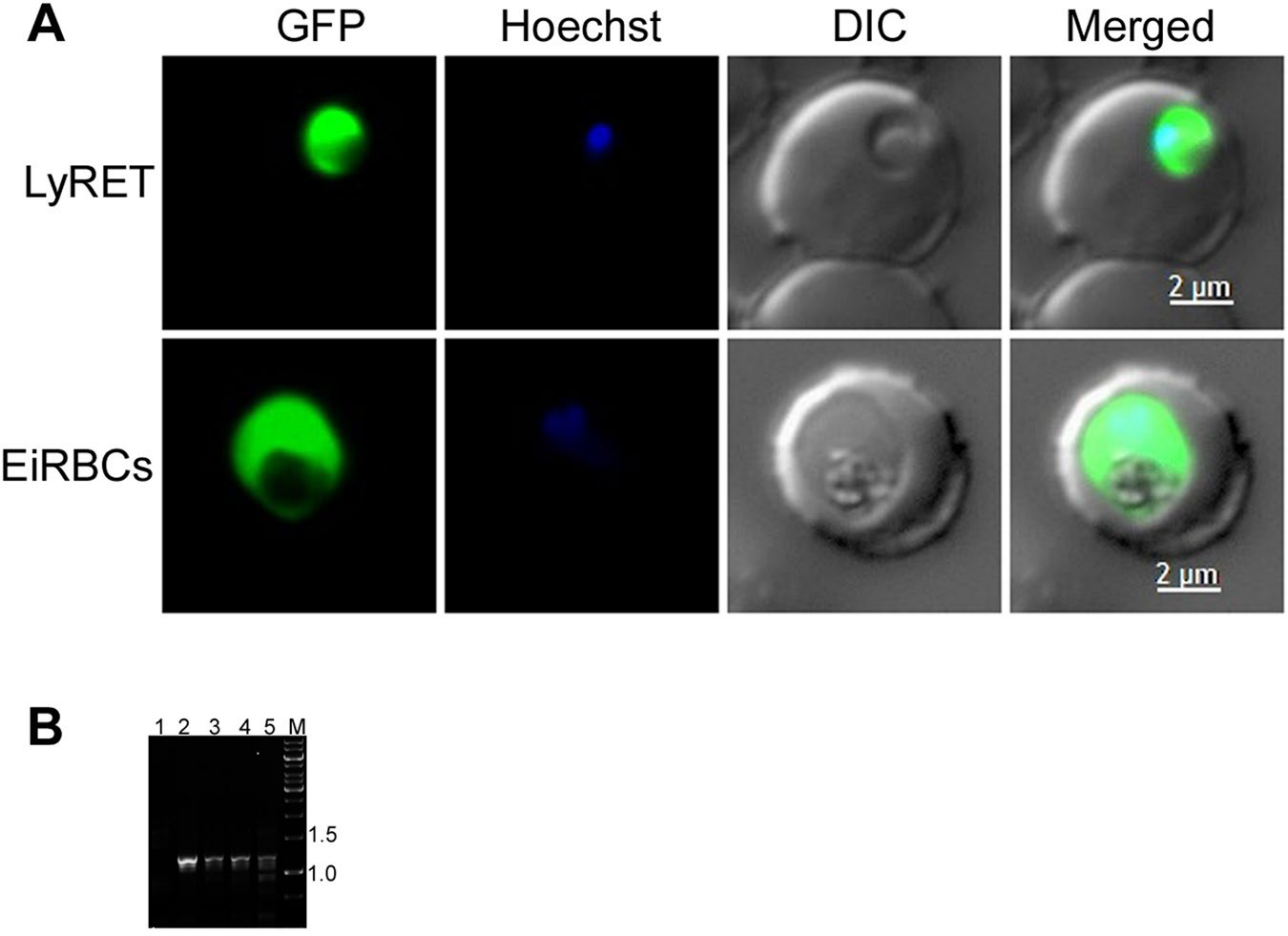
(**A**) GFP expression in drug resistant parasites. *P. falciparum* 3D7 was transfected with pFCEN1 using LyRET and EiRBC methods. Drug resistant parasites were evaluated for GFP expression using live cell fluorescence microscopy. Shown are images for GFP expression (GFP), nuclear stain (Hoechst), bright field (DIC) and the overlap of all images (Merged). (**B**) The presence of HFDDI in parasites. *P. falciparum* 3D7 was transfected with HFDDI using LyRET and EiRBC methods. The presence of HFDDI in drug resistant parasites was determined by PCR amplification of *P. yoelii* α-tubulin region of the plasmid from the total DNA. The ethidium bromide stained agarose gel shows PCR products amplified from the total DNAs of parasites obtained by LyRET (lanes 3 and 5) and EiRBC (lanes 2 and 4) methods. Genomic DNA of wild type parasites served as a negative control (lane 1). The lane M contains DNA size markers with the positions of 1.5 kbp and 1.0 kbp markers indicated.

Since LREs efficiently supported invasion and development of *P. falciparum*, we assessed DNA-containing LREs for transfection method (LyRET) with *P. falciparum* 3D7, and compared its success rate with the success rates of electroporation-based methods involving electroporation of DNA into uninfected RBCs (Epreloading) and ring stage-infected RBCs (EiRBC). We used pPfCENv3 for most of the experiments. The transfection success rates of LyRET and EiRBC methods were similar and better than Epreloading method (Table 1). Drug resistant parasites emerged in 11–14 days in all three methods, which expressed GFP (Fig. 2A). We also assessed the LyRET method with the *P. falciparum* D10 strain using pPfCENv3. Drug resistant D10 parasites emerged in 2 weeks, which also expressed GFP (Fig. 2B). We next compared LyRET and EiRBC methods with *P. falciparum* 3D7 using the plasmids HFDDI and pFCEN1, which allow selection with WR99210. Both the methods yielded drug resistant parasites, albeit with a slightly better success rate in case of EiRBC method (Table 1). Parasites containing the pFCEN1 plasmid expressed GFP (Fig. 3A), indicating that the drug resistant parasites are truly recombinant parasites. PCR of the total DNA of HFDDI-transfected parasites amplified *P. yoelii* α-tubulin region of the plasmid, indicating that the parasites are recombinant (Fig. 3B).

**Table 1:** Comparison of the success rate of transfection of *P. falciparum* by LyRET, Epreloading, and EiRBC methods.

Plasmids	Transfection methods used for *P. falciparum* 3D7					
	LyRET		EiRBC		Epreloading	
	No. of successful transfections/total No. of transfections done	Days to emergence of resistant parasites	No. of successful transfections/total No. of transfections done	Days to emergence of resistant parasites	No. of successful transfections/total No. of transfections done	Days to emergence of resistant parasites
pPfCENv3	8/8	11 ± 0.9	5/5	11.4 ± 0.5	3/4	14 ± 0
HFDDI	7/8	37.3 ± 5.8	4/4	26.5 ± 3.5	*NA	NA
pFCEN1	1/2	46	2/2	23 ± 0	NA	NA
	**Transfection methods used for** ***P. falciparum*** **D10**					
pPfCENv3	6/6	14 ± 1.6	NA	NA	NA	NA

Each transfection experiment was independently performed. The days to emergence of resistant parasites is mean with SD of the days required for emergence of resistant parasites in successful experiments. *NA indicates “Not Attempted”.

In transfections by both LyRET and EiRBC methods, the emergence of resistant parasites was significantly delayed in case of HFDDI and pFCEN1 compared to those with pPfCENv3 (Table 1). This delay could be due to difference in the expression levels of selection markers (BSD in pPfCENv3 versus hDHFR in HFDDI and pFCEN1), as the selection marker genes are under different regulatory sequences (Supplementary Information). Whether this delay is also attributed to the drug used for selection remains to be tested. One can test this using plasmids containing BSD and hDHFR genes under identical regulatory elements. Compared to the EiRBC method, there was a 1.5 to 2-fold delay in emergence of resistant parasites in transfections by the LyRET method with HFDDI and pFCEN1 plasmids. We speculate that this difference may be due to the potential of EiRBC method to electroporate some DNA molecules directly into the parasite, whereas parasites have to take up DNA from the RBC cytosol in case of LyRET. The different mode of introducing DNA by LyRET and EiRBC methods together with the intrinsic nature of HFDDI and pFCEN1 plasmids might have been responsible for the overall delay.

Most of the transfection experiments with *P. falciparum* require 50–100 µg plasmid DNA/transfection. Hence, we tested a range of plasmid DNA amounts (1 to 100 μg/transfection) to determine the minimum plasmid DNA amount for successful transfection by the LyRET method. Drug resistant parasites emerged in 12–24 days with 2.5–100 μg plasmid DNA/transfection (Table 2), and the parasites from these experiments showed GFP fluorescence in live cell microscopy (data not shown). Transfections without any plasmid DNA or with 1.0 μg plasmid DNA/transfection yielded unstable drug resistant parasites, which did not show GFP and were eliminated at higher selection pressure, indicating that these were untransfected parasites (Table 2). 2.5 μg is the minimum amount of plasmid DNA for successful transfection by the LyRET method. However, resistant parasites emerged almost 12 days earlier in transfections with ≥40 μg plasmid DNA as compared to transfections with 2.5 μg plasmid DNA. Hence, 40 μg of plasmid DNA appears to be optimum amount for transfection by the LyRET method.

**Table 2:** Optimization of plasmid DNA amount for transfection of *P. falciparum* 3D7 by the LyRET method.

μg of pPfCENv3/transfection	No. of successful transfections/total No. of transfections done	Days to emergence of resistant parasites
0	0/2	—
1	0/2	—
2.5	2/2	24 ± 1.4
5	2/2	23 ± 0
10	6/6	17 ± 4.7
20	4/4	16 ± 1.2
40	4/4	12.5 ± 1.9
80	3/3	13 ± 2
100	4/4	12 ± 2

Each transfection experiment was independently performed. The days to emergence of resistant parasites is mean with SD of the days required for emergence of resistant parasites in successful experiments.

As *P. falciparum* strains have been shown to invade RBCs of different blood groups with different efficiency in *in vitro* conditions^25^, we assessed the LyRET method for transfection of RBCs of different blood groups. Recombinant parasites were obtained with RBCs of all the blood groups, which, however, differed in days required to reach 1–2% parasitemia (Table 3). Transfections of blood group O by the LyRET method was the most efficient in terms of the number of days required to reach 1–2% parasitemia and %GFP population, and it was comparable with the EiRBC method for the same blood group (Table 3). Live fluorescence microscopy of recombinant parasites from all the transfection experiments indicated GFP expression (data not shown). The better transfection efficiency with O blood group RBCs could be due to more efficient invasion of this blood group of RBCs by *P. falciparum*, as has been reported previously^25^. RBCs of different blood groups may affect parasite development and egress of merozoites, which could determine transfection efficiency. Whether the blood group O would also result in the best transfection efficiency by EiRBC method should be tested. Nonetheless, our transfection results support the use of O blood group RBCs for transfection of *P. falciparum* by the LyRET method.

**Table 3:** Transfection of *P. falciparum* 3D7 with pPfCENv3 using RBCs of different blood groups by the LyRET method.

Blood groups	LyRET			EiRBC		
	No. of successful transfections/total No. of transfections done	Days to reach 1–2% parasitemia	%GFP positive cells	No. of successful transfections/total No. of transfections done	Days to reach 1–2% parasitemia	%GFP positive cells
O+	4/4	20.0 ± 0.0	53.1 ± 8.5	4/4	19.5 ± 0.7	49.2 ± 6.6
A+	4/4	35.0 ± 9.9	32.2 ± 3.1	*NA	NA	NA
B+	4/4	20.5 ± 0.7	48.3 ± 24.5	NA	NA	NA
AB+	4/4	34.0 ± 11.3	34.3 ± 11.7	NA	NA	NA

Each transfection experiment was independently performed. The days to reach 1–2% parasitemia is mean with SD of the days required for emergence of resistant parasites in successful experiments. The %GFP positive cells was determined by FACS and is mean with SD of GFP-Hoechst positive cells in all the successful experiments. *NA indicates “Not Attempted”.

The LyRET method resulted in successful transfection of different *P. falciparum* strains with different plasmid DNAs, which indicate broad applicability of this newly developed method. Of the 65 transfections carried out by the LyRET method (using 2.5–100 µg plasmid DNA), recombinant parasites were obtained in 63 cases. Since the LyRET method yielded successful transfection with as little as 2.5 µg plasmid DNA and the incorporation of DNA into RBCs does not involve any additional steps, it can be adapted to a high throughput method. Minor optimization might be required to adapt this method to other *Plasmodium* species. Furthermore, we show that RBCs of O blood group are most suitable for transfection by the LyRET method. While the overall success rate of LyRET method is comparable with the EiRBC method, the simplicity and economic aspects make the LyRET method an alternative to the current transfection methods for *P. falciparum*.

## Supplementary information

Supplementary information 

## References

[1] Goonewardene R (1993). Transfection of the malaria parasite and expression of firefly luciferase. Proceedings of the National Academy of Sciences.

[2] van Dijk MR, Waters AP, Janse CJ (1995). Stable transfection of malaria parasite blood stages. Science.

[3] Wu Y, Sifri CD, Lei HH, Su XZ, Wellems TE (1995). Transfection of *Plasmodium falciparum* within human red blood cells. Proc Natl Acad Sci USA.

[4] Wu Y, Kirkman LA, Wellems TE (1996). Transformation of *Plasmodium falciparum* malaria parasites by homologous integration of plasmids that confer resistance to pyrimethamine. Proc Natl Acad Sci USA.

[5] Fidock DA, Wellems TE (1997). Transformation with human dihydrofolate reductase renders malaria parasites insensitive to WR99210 but does not affect the intrinsic activity of proguanil. Proc Natl Acad Sci USA.

[6] Deitsch K, Driskill C, Wellems T (2001). Transformation of malaria parasites by the spontaneous uptake and expression of DNA from human erythrocytes. Nucleic Acids Res.

[7] Mamoun CB (1999). Transfer of genes into *Plasmodium falciparum* by polyamidoamine dendrimers. Mol Biochem Parasitol.

[8] Gopalakrishnan AM, Kundu AK, Mandal TK, Kumar N (2013). Novel nanosomes for gene delivery to *Plasmodium falciparum*-infected red blood cells. Sci Rep.

[9] Fotoran WL, Santangelo R, de Miranda BNM, Irvine DJ, Wunderlich G (2017). DNA-Loaded Cationic Liposomes Efficiently Function as a Vaccine against Malarial Proteins. Mol Ther Methods Clin Dev.

[10] Janse CJ (2006). High efficiency transfection of *Plasmodium berghei* facilitates novel selection procedures. Molecular and Biochemical Parasitology.

[11] Janse CJ, Ramesar J, Waters AP (2006). High-efficiency transfection and drug selection of genetically transformed blood stages of the rodent malaria parasite *Plasmodium berghei*. Nature Protocols.

[12] Caro F, Miller MG, DeRisi JL (2012). Plate-based transfection and culturing technique for genetic manipulation of *Plasmodium falciparum*. Malaria journal.

[13] Bushell E (2017). Functional Profiling of a Plasmodium Genome Reveals an Abundance of Essential Genes. Cell.

[14] Zhang Min, Wang Chengqi, Otto Thomas D., Oberstaller Jenna, Liao Xiangyun, Adapa Swamy R., Udenze Kenneth, Bronner Iraad F., Casandra Deborah, Mayho Matthew, Brown Jacqueline, Li Suzanne, Swanson Justin, Rayner Julian C., Jiang Rays H. Y., Adams John H. (2018). Uncovering the essential genes of the human malaria parasitePlasmodium falciparumby saturation mutagenesis. Science.

[15] Murphy SC (2006). Erythrocyte G protein as a novel target for malarial chemotherapy. PLoS Med.

[16] Frankland S (2006). Delivery of the malaria virulence protein PfEMP1 to the erythrocyte surface requires cholesterol-rich domains. Eukaryot Cell.

[17] Chandramohanadas R (2009). Apicomplexan parasites co-opt host calpains to facilitate their escape from infected cells. Science.

[18] Abu Bakar N, Klonis N, Hanssen E, Chan C, Tilley L (2010). Digestive-vacuole genesis and endocytic processes in the early intraerythrocytic stages of *Plasmodium falciparum*. J Cell Sci.

[19] Trager W, Jensen JB (1976). Human malaria parasites in continuous culture. Science.

[20] Lambros C, Vanderberg JP (1979). Synchronization of *Plasmodium falciparum* erythrocytic stages in culture. J Parasitol.

[21] Iwanaga S, Kato T, Kaneko I, Yuda M (2012). Centromere Plasmid: A New Genetic Tool for the Study of *Plasmodium falciparum*. PLOS ONE.

[22] Sijwali PS, Rosenthal PJ (2010). Functional evaluation of Plasmodium export signals in *Plasmodium berghei* suggests multiple modes of protein export. PLoS One.

[23] Singhal N, Atul, Mastan BS, Kumar KA, Sijwali PS (2014). Genetic ablation of plasmoDJ1, a multi-activity enzyme, attenuates parasite virulence and reduces oocyst production. Biochem J.

[24] Sijwali PS, Koo J, Singh N, Rosenthal PJ (2006). Gene disruptions demonstrate independent roles for the four falcipain cysteine proteases of *Plasmodium falciparum*. Mol Biochem Parasitol.

[25] Theron M, Cross N, Cawkill P, Bustamante LY, Rayner JC (2018). An *in vitro* erythrocyte preference assay reveals that *Plasmodium falciparum* parasites prefer Type O over Type A erythrocytes. Scientific Reports.

