# Supplementary material for: Lyse-Reseal Erythrocytes for Transfection of Plasmodium falciparum
Source: Sci Rep. 2019 Dec 27;9:19952. doi: 10.1038/s41598-019-56513-9 (PMC6934678; doi:10.1038/s41598-019-56513-9)

**Lyse-Reseal Erythrocytes for Transfection of *Plasmodium falciparum***

Gokulapriya Govindarajalu, Zeba Rizvi, Deepak Kumar and Puran Singh Sijwali*

**Requirement**

- *Plasmodium falciparum* strains
- Fresh human erythrocytes
- Gas mixture (5% CO_2_, 5% O_2_, and 90% N_2_)
- Parasite culture medium: RPMI 1640 containing 2 g/l sodium bicarbonate, 2 g/l glucose, 25 µg/ml gentamicin, 300 mg/l glutamine, 0.5% albumax II
- RBC storage medium: RPMI 1640 with 2 g/l glucose and 25 µg/ml gentamicin
- 5% D-sorbitol (in distilled water)
- RBC lysis buffer: 5 mM K_2_HPO_4_, 1 mM ATP, pH 7.4
- Resealing solution stocks: 5 M NaCl, 1 M MgCl_2_, 100 mM ATP, 100 mM GSH
- Cytomix: 10 mM K_2_HPO_4_-KH_2_PO_4_, 120 mM KCl, 0.15 mM CaCl_2_.2H_2_O, 25 mM HEPES, 2 mM EGTA, 5 mM MgCl_2_.6H_2_O, pH 7.6
- Phosphate Buffered Saline (PBS)
- 65% Nycodenz density gradient solution (in 1x PBS)
- Maxi plasmid preparation kit
- Cell culture plastic ware (pipettes, centrifuge tubes and culture flasks)
- Centrifuge equipped with swinging-bucket rotors for 15 and 50 ml conical centrifuge tubes
- Centrifuge equipped with fixed-angle rotors for 1.5 ml micro centrifuge tubes
- 37°C Incubator
- Biosafety laminar hood
- Giemsa stain

**STEPWISE PROTOCOL**

**Day before the transfection**

1. Synchronize a *P. falciparum* culture when the majority of parasites (>10%) are at ring stage as follows
   1. Centrifuge 25-30 ml of the culture at 662xg using a swinging-bucket rotor for 5 min at room temperature.
   2. Aspirate the supernatant, resuspend the pellet in 10x (packed cell volume or PCV) of 5% D-sorbitol and incubate at 37°C for 10 min with intermittent shaking.
   3. Centrifuge the sample for 5 min at room temperature at 662xg using a swinging-bucket rotor.
   4. Aspirate the supernatant and resuspend the pellet in 25-30 ml of parasite culture medium. Transfer the culture to a 75 cc flask and grow under the standard culture conditions for *P. falciparum*.

**Day 0 (transfection day)**

1. Collect blood from human volunteers by venipuncture in heparin- or EDTA-containing tubes.
2. Centrifuge the blood at 662xg using a swinging-bucket rotor for 5 min at room temperature. Remove plasma and buffy coat carefully by aspiration. Estimate PCV of the RBC pellet.
3. Resuspend the pellet in 2x PCV of the RBC storage medium and transfer to a 15 ml conical centrifuge tube. Centrifuge the suspension at 662xg for 5 min at room temperature using a swinging-bucket rotor. Repeat this step one more time.
4. Resuspend the pellet in equal volume of the RBC storage medium (50% hematocrit) and keep at 4°C until used.

**Preparation of lyse-reseal erythrocytes (LREs)**

Use 200 μl RBC suspension (~100 μl PCV) for one transfection

1. Transfer 200 μl RBC suspension (prepared in step 5) into a 1.5 ml MCT, centrifuge at 371xg for 5 min at 4°C using a fixed-angle rotor and remove the supernatant.
2. Resuspend the pellet (~100 μl PCV) in 10x PCV of ice cold PBS, centrifuge at 371xg for 5 min at 4°C using a fixed-angle rotor, and remove the supernatant. Repeat this step one more time.
3. Resuspend the RBC pellet in equal volume of ice cold RBC lysis buffer containing 100 μg plasmid DNA. Incubate at 4°C for 1 hour with gentle rotation.
4. Estimate the volume of RBC ghost suspension. Add to it appropriate volumes of resealing solution stocks to achieve the resealing buffer concentration (150 mM NaCl, 5 mM MgCl_2_, 1 mM ATP and 1 mM GSH). Incubate the suspension at 37°C for 1 hour with shaking at 55 rpm to allow resealing of RBC ghost.
5. Transfer the suspension to a 15 ml conical centrifuge tube, add 10 ml RBC storage medium (prewarmed to 37°C) to it, centrifuge at 662xg using a swinging-bucket rotor at room temperature and remove the supernatant. Repeat this step one more time.
6. Finally, resuspend the DNA-encapsulated lyse-reseal erythrocyte (LRE) pellet in equal volume of RBC storage medium for subsequent use.

**Purification of late trophozoite/schizont stage parasites**

1. Transfer 25-30 ml culture with mostly late trophozoite/schizont stages to a 50 ml conical centrifuge tube (from step 1). Carefully add 10 ml of 65% nycodenz density gradient solution underneath the culture. Centrifuge at 360xg for 15 min at 25°C using a swinging-bucket rotor (with maximum acceleration and zero deceleration).
2. Transfer the interphase containing infected RBCs with a pipette to a 15 ml conical centrifuge tube. Centrifuge the sample at 662xg for 5 min at room temperature using a swinging-bucket rotor. Remove the supernatant by aspiration.
3. Resuspend the pellet in 10 ml parasite culture medium (prewarmed to 37°C), centrifuge the sample at 662xg for 5 min at room temperature using a swinging-bucket rotor and discard the supernatant by aspiration. Repeat this step one more time.
4. Resuspend the pellet in 1 ml parasite culture medium. Process a 50 µl aliquot of the sample for Giemsa smear to assess purity of the sample. The purified sample should contain >80% late trophozoite/schizont-infected RBCs.

**Infection of LREs**

1. Transfer the LREs suspension (prepared in step 11) to a 25 cc flask containing 5 ml parasite culture medium (to achieve 2% hematocrit).
2. Add purified trophozoite/schizont-infected RBC suspension to achieve 2-3% parasitemia. Gas the flask and follow standard culture conditions for *P. falciparum*.

**Culture maintenance**

1. On day 1, prepare a Giemsa smear to estimate parasitemia (most of the iRBCs are expected to have rings). Change the culture medium.
2. On day 2, prepare a Giemsa smear to estimate parasitemia. If required, expand the culture to 10 ml with fresh normal RBCs to achieve 4-5% parasitemia.
3. On day 3, change the culture medium and start selection by adding the appropriate drug (blasticidin: 1 μg/ml, WR99210: 0.5-1 nM).
4. Continue culturing in the presence of drug for 5 cycles, followed by without drug for 3 cycles, and thereafter in the presence of drug for the desired period.

**Note:**

- One should use fresh RBCs for transfection experiments, as the initial RBCs used in transfection experiments remain in the culture for about a month. We have used upto 3 days old blood without any noticeable effect on transfection success.
- The culture medium should be changed every day for the first week, followed by on alternate days. 50 μl suspension of fresh normal RBCs needs to be added to the culture once a week to replenish old and lysed RBCs.
- Routinely monitor the culture for parasites by observing Giemsa smears. Upon emergence of recombinant parasites, expand the culture and process for downstream experiments as desired (preparation of frozen stocks and evaluation of recombinant parasites).
- All the solutions must be sterilized using appropriate methods. Sterile conditions must be followed in performing all the steps.

**Transfection plasmids used in the study.** The regulatory and coding regions are labeled. Arrows indicate 5'-3' direction of the corresponding region, and unique restriction sites are indicated with their positions in the plasmid. pPfCENv3 (8553 bps) contains the BSD gene under PcDT 5’U and PfHrp 3’U regulatory elements for selection of transfected parasites with blasticidin. Transcription of GFP gene is driven by PfCam 5’U and PfHsp86 3’U elements. pFCEN1 (8018 bps) contains hDHFR gene under Pbef and PbDT 3’U regulatory elements for selection of transfected parasites with WR99210. Pbef also functions as a bifunctional promoter for GFP with PbHSP 3’U as a transcription terminator. HFDDI (8113 bps) contains hDHFR gene under PyaTbl 5’U and PfHrpII 3’U regulatory elements for selection of transfected parasites with WR99210. The Pf centromeric sequence in pPfCENv3 and pFCEN1 allows maintenance of these plasmids as a single copy in the parasite, whereas the ampicillin cassette in all plasmids allows their maintenance in bacteria.


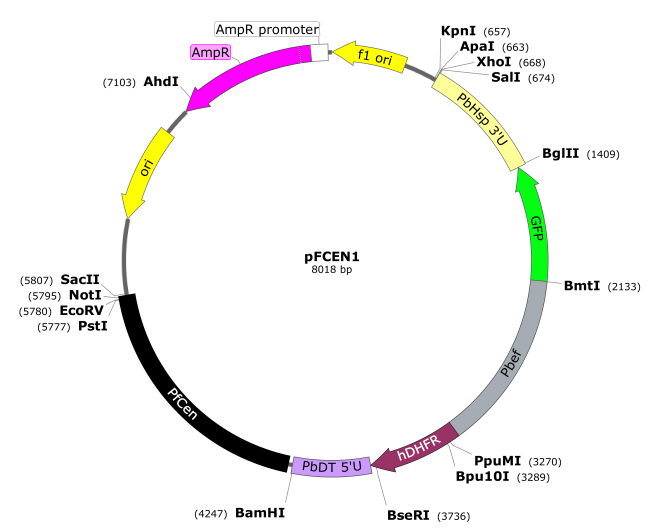

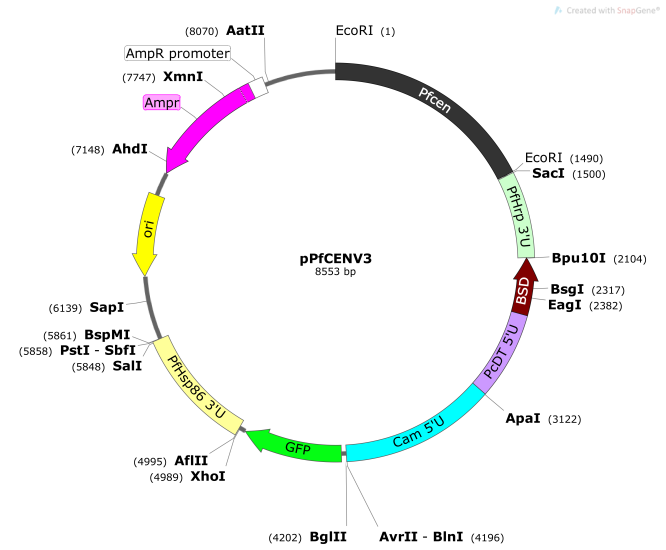

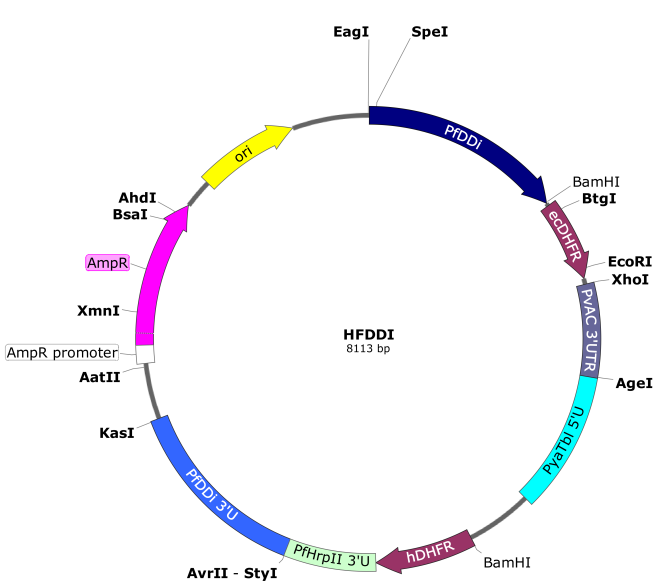

Supplement: Supplementary file 1 — Supplementary information [file 41598_2019_56513_MOESM1_ESM.docx]
